# Research on the Performance and Application of High-Performance PE Composite Modified Asphalt

**DOI:** 10.3390/polym17030346

**Published:** 2025-01-27

**Authors:** Lei Xia, Qidong Su, Xiaolong Yang, Shixi Lin, Haoran Wang, Rongguo Hou, Dongwei Cao

**Affiliations:** 1 School of Materials Science and Engineering, Chang’an University, Xi’an 710061, China; 2 China-Road Transportation Verification & Inspection Hi-Tech Co., Ltd., Beijing 100088, China; 3 Research Institute of Highway Ministry of Transport, Beijing 100088, Chinarg.hou@rioh.cn (R.H.); 4 Cangzhou Qugang Expressway Construction Co., Ltd., Cangzhou 062450, China

**Keywords:** PE composite modified asphalt, storage stability, anti-aging performance, microscopic properties

## Abstract

The large-scale production of waste plastics has brought serious environmental pollution problems and its recycling and high value-added utilization technology remains a global challenge. Therefore, this study uses waste polyethylene (PE) to prepare high-performance polyethylene composite modified asphalt (HPEA), solving the problem of poor stability and low temperature performance of traditional plastic modified asphalt, while achieving high value-added utilization of waste plastics. A high-performance polyethylene composite modifier (HPE) was prepared through mechanochemical and thermochemical interactions. Then HPEA with different HPE content and styrene-butadiene-styrene (SBS) modified asphalt (SBSMA) with different SBS content were prepared. Compare and analyze the conventional performance, storage stability, anti-aging performance and microscopic properties of HPEA and SBSMA. The results are as follows: (1) the conventional performance of HPEA is comparable to, or superior to, that of SBSMA. The addition of HPE resulted in a significant decrease in asphalt penetration. The modification effect achieved by adding 3–5% SBS to Kunlun 70# asphalt is equivalent to that achieved by incorporating 4–6% HPE. (2) HEPA exhibits good storage stability and no obvious segregation phenomenon. When the HPE content changes from 4% to 8%, the maximum difference in 48 h softening point of HPEA is 1.1 °C, which is significantly smaller than the 48 h softening point difference of SBSMA when the SBS content changes from 3% to 5%. (3) When HPE attains a specific concentration, HPEA can exhibit an anti-aging performance that is comparable to, or superior to, that of SBSMA. (4) The infrared spectrum of HPEA closely resembles that of SK70# matrix asphalt. The modification of HPEA primarily involves physical blending, with HPE undergoing development and re-crosslinking within the system, leading to interactions between smaller particles and asphalt, resulting in the formation of a relatively stable three-dimensional spatial structure.

## 1. Introduction

With the development of highway engineering construction, asphalt pavement has become the main form for road traffic. At present, highways generally have a high traffic volume and a large number of heavy-duty vehicles, resulting in a significant reduction in the actual service life of asphalt pavement [1,2]. In asphalt mixtures, asphalt plays an important role as a binder in road performance and service life, so high-performance asphalt is needed to improve the service performance of the road surface [3]. Research has shown that polymers can improve asphalt performance to a certain extent and have been widely validated in practice. However, common polymer modifiers such as styrene-butadiene-styrene (SBS), styrene butadiene rubber (SBR), ethylene vinyl acetate copolymer (EVA), and polypropylene (PP) have problems such as high cost, poor compatibility, and high construction temperature [4,5]. Researchers have attempted to address the drawbacks of polymer modified asphalt by using functionalized polymers or additional additives such as sulfur, antioxidants, and hydrophobic clay minerals [6,7,8]. Although these solutions have made up for some of the shortcomings of polymer modified asphalt, they have caused many operational problems in engineering applications, such as storage stability issues caused by differences in physical properties between polymers and asphalt, and cost issues caused by high prices of modifiers [9,10]. Therefore, the synthesis of asphalt polymer modifiers with excellent performance, economy, and environmental friendliness not only has high theoretical research value, but also has extensive engineering application value.

The emergence of plastics has created many technologies and products that have driven the development of human society and improved living standards. However, due to the inherent difficulty of plastic degradation, it has brought many adverse effects on the ecological environment of the earth [11,12]. The Earth has produced over 9 billion tons of plastic waste to date, of which only 9% can be recycled and reused, with the majority being disposed of in landfills or at sea [13]. The total amount of discarded plastic products every year is enormous, posing a great challenge to human survival. Plastics, also known as high molecular weight polymers, are generally divided into thermosetting plastics and thermoplastic plastics [14]. Plastic products are extremely difficult to degrade and require over 500 years to degrade in soil [15,16]. Research has shown that the physical and chemical properties of plastics are stable and can be used for the modification of road asphalt [17,18]. At present, the plastics mainly used for asphalt modification include polyethylene (PE), polypropylene (PP), polystyrene (PS), and ethylene vinyl acetate copolymer (EVA) [19,20,21]. In recent years, research on the application of waste plastics in asphalt road engineering has gradually increased, becoming a hot topic in the recycling and utilization of waste resources [22,23,24,25].

Polyethylene is a polymer compound formed by the polymerization of ethylene molecules and is currently one of the most widely used plastics in the world. Polyethylene is divided into low-density polyethylene (LDPE), high-density polyethylene (HDPE), and linear low-density polyethylene (LLDPE) [26]. Due to its relatively low cost, as early as 1983, the Transportation and Road Research Laboratory in the United States began systematic research on a polyethylene modified asphalt called Novophalt and the results showed that polyethylene could significantly improve the road performance of base asphalt. Liang modified the matrix asphalt by compounding SBS and EVA with PE to produce an asphalt mixture called Polyphalt and found that PE can exist relatively stably in Polyphalt [27]. Considering the high production and consumption of PE in daily life, road engineers in many countries have started using native and recycled PE materials for asphalt modification [28,29]. 

Polyethylene can change the glass transition temperature of blends and crystallize from the blend during cooling to increase the stiffness of asphalt until the crystals melt [30]. The addition of all types of PE will have an impact on the road performance of asphalt. Liang studied the phase behavior of PE in PE modified asphalt through experimental and simulation methods. The results showed that after the decrease of PE density and crystallinity, the asphalt component was more likely to migrate to the intermolecular space, and the interaction between the two was stronger, resulting in higher swelling degree [27]. Polyolefin interacts with the lightweight components of asphalt, forming a biphasic structure of polyolefin phase and asphalt phase in the asphalt matrix. According to current research, PE modified asphalt and asphalt mixtures have good fatigue resistance, high temperature stability, and water damage resistance [31]. Dalhat’s research found that PE modified asphalt has a lower irreversible creep compliance value, indicating good high-temperature resistance to rutting [28,32]. Habib conducted research on LLDPE and HDPE modified asphalt and found that thermoplastic copolymers can significantly reduce the penetration of matrix asphalt. The best modification effect is achieved when the polyethylene content is kept below 3 wt% [33].

Although PE can significantly improve the high-temperature performance of asphalt, the low-temperature performance of PE modified asphalt has always been a controversial issue. Some studies suggest that the addition of PE does not improve the low-temperature performance of asphalt [34]. Du conducted a study on the low-temperature performance of different types of polymer modified asphalt using a ductility meter and a bending beam rheometer (BBR). The results indicate that the low-temperature performance of PE modified asphalt is lower than that of SBS modified asphalt and EVA modified asphalt [35]. Firoozifar modified the matrix asphalt with three types of polyethylene (HDPE, LDPE, LLDPE) and found that the low-temperature ductility of different types of PE modified asphalt varied greatly. The low-temperature performance of LDPE modified asphalt is superior to that of HDPE and LLDPE modified asphalt [36].

In early research, the surface of LDPE modified asphalt exhibited a rough and uneven appearance. In addition, researchers have explored the micro interactions between PE and asphalt and found that there are issues with phase separation and storage stability in PE modified asphalt [33]. The incompatibility between polymer phase and asphalt phase can lead to phase separation at high temperatures. From a dynamic perspective, the system of polymer and asphalt is stable at low temperatures. However, when the temperature rises above the critical point, the polymer and asphalt system tend to separate [37,38]. PE should be fully compatible with asphalt to create a uniform binder and minimize phase separation issues during storage, transportation, and use, thereby ensuring the road performance of polyethylene modified asphalt. Due to the significant differences in molecular weight, structure, viscosity, and density between PE and asphalt components, it is difficult to achieve compatibility between polyethylene and asphalt. Therefore, solving the storage stability problem of polyethylene modified asphalt is very important [39]. In current research, in addition to functional modifiers used to improve the compatibility between PE and asphalt, additives such as montmorillonite, crosslinking agents, sulfur, and silica have also been used to enhance the compatibility between polyethylene and asphalt [17].

The main purpose of this study is to address the issues of poor low-temperature performance and storage stability of PE modified asphalt and to investigate the modification mechanism of PE modified asphalt. A high-performance polyethylene (PE) composite modifier (HPE) was prepared through mechanochemical and thermochemical reactions. Then high-performance PE composite modified asphalt (HPEA) with different HPE content and SBS modified asphalt (SBSMA) with different SBS content were prepared. The conventional performance, storage stability, and anti-aging performance of HPEA and SBSMA were compared and analyzed and the modification mechanism of HPEA was analyzed through microscopic test. Finally, excellent performance polyethylene modified asphalt is obtained, which solves the problems of poor storage stability and low-temperature performance of traditional polyethylene modified asphalt and achieves high value-added utilization of waste plastics.

## 2. Materials and Methods

### 2.1. Raw Materials

In this paper, the 70# matrix asphalt was produced by China National Offshore Oil Corporation in Qinhuangdao of China. Its technical indicators are in line with the standards of heavy asphalt 70# in the current national standard “Technical Specification for Highway Asphalt Pavement Construction” (JTG F40-2004) [40].Table 1 below shows the test results and technical indicators of the matrix asphalt.

The SBS model used in this study is SBS1301, a linear type produced by Yueyang Petrochemical Plant in Yuyang of China. The basic properties of SBS are detailed in Table 2.

Waste plastics are used with the largest amount of waste polyethylene at present and the melting index is 1.5 g/10 min (230 °C, 2.16 kg). The waste polyethylene was procured from Sinopec Beijing Yanshan Petrochemical Company in Beijing of China.

The plasticizer used in the study is Dioctyl Phthalate liquid sourced from Henan Leimo Chemical Products Co., Ltd. in Zhengzhou of China. The stabilizer is a composite stabilizer mixed with elemental sulfur and a commercially available modified asphalt stabilizer and a homemade stabilizer. In this paper, the stabilizer content is 1.5‰ (accounting for asphalt content). The solubilizer is rubber oils rich in aromatics procured from Shandong Hengfeng Rubber Powder Co., Ltd. in Shandong of China.

### 2.2. Test Methods

#### 2.2.1. Conventional Performance Test of Modified Asphalt

This study evaluated the performance indicators of asphalt, including 25 °C penetration, softening point, 5 °C ductility.

The penetration index is the depth (1/10 mm is one degree) of the penetration into the asphalt sample within the specified time 5 s with the standard needle with the specified mass of 100 g at the specified temperature of 25 °C.

The softening point index is determined by a global method. The asphalt sample is loaded into the copper ring of the specified size (diameter 16 mm, height 6 mm) and a standard steel ball is placed on the sample, which is immersed in water and heated at the specified speed (5 °C/min) to make the asphalt soften and sag. When the sag reaches 25.4 mm, the temperature is the softening point.

The ductility index is measured by the ductility meter, the asphalt sample is made into a standard test mold, and the elongation length is expressed in cm when it is broken under the specified tensile speed (5 cm/min) and the specified temperature (25 °C). The larger the ductility value, the better the ductility.

#### 2.2.2. Storage Stability Test

Storage stability is a key assessment index of asphalt quality. The 48 h softening point difference between upper and lower layers of asphalt is commonly used to evaluate the stability of asphalt. In practical engineering applications, due to the influence of admixtures, some of the indicators will change to different degrees from the end of production and processing to the end of use.

#### 2.2.3. Thin Film Oven Test (TFOT)

In the process of this research, firstly, the asphalt binder is taken as the starting point and the modified asphalt is heated at high temperature at 163 °C by using a thin film oven, and the aging asphalt is tested.

#### 2.2.4. Microscopic Analysis of Modified Asphalt

(1) Fluorescence microscopic test

The asphalt was analyzed using an infrared spectrometer. In the modified asphalt, when the modifier is irradiated by the blue light source emitted by the fluorescence microscope, it presents a longer wavelength of yellow light, while the matrix asphalt presents a much dimmer image under the blue light source. Therefore, the microscopic morphology of modified asphalt and the dissolution of modifier in asphalt can be clearly seen by taking pictures of modified asphalt with a fluorescence microscope [41].

(2) Infrared spectrum test

On the basis of fluorescence microanalysis, the matrix asphalt and high performance waste plastic composite modified asphalt were analyzed with a Fourier transform infrared spectrometer (FTIR). The molecular structure and chemical groups of modified asphalt were deduced from the absorption frequency and absorption peak intensity of infrared spectrum and the qualitative analysis of high performance waste plastic composite modified asphalt material was completed [42,43]. The instrument used is the TENSOR27 FTIR Fourier transform infrared spectrometer produced by Colliers of the United States.

### 2.3. Preparation Process of PE Composite Modifier and Modified Asphalt

**Figure 1:**
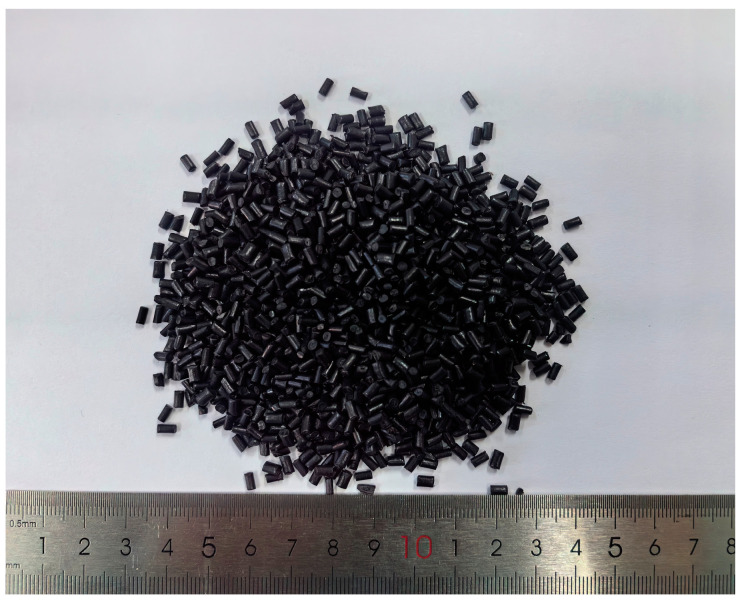
The high-performance composite modifier particles.

An SHJ-20 twin-screw extruder produced by Nanjing Hanyi Machinery Co., Ltd. in Nanjing of China is selected as the equipment for processing high performance composite modifiers for waste plastics. A certain proportion of waste plastic high density polyethylene (HDPE), SMN resin, diatomite, ethylene-vinyl acetate copolymer (EVA), JC-GJ301 nano reinforcer, and an additive are mixed in a high speed mixer and then the reactive blending process is carried out by a twin-screw extruder. High performance composite modifier particles of waste plastics were prepared by extrusion granulation. The high-performance composite modifier particles of waste plastics are shown in Figure 1.

**Figure 2:**
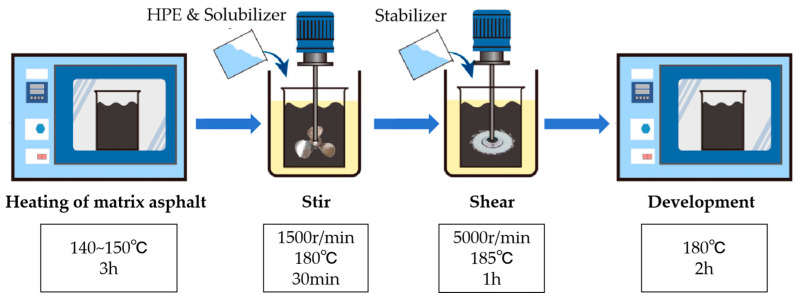
Preparation process of high-performance PE composite modified asphalt.

High speed shear machines and mixers are used to prepare high performance waste plastic composite modified asphalt. First, the high performance waste plastic composite modifier is stirred in the asphalt to swell and then a certain amount of stabilizer is added after cutting with a high speed shear machine. Finally, the development is completed in the oven. The preparation process of high performance waste plastic composite modified asphalt is shown in Figure 2.

The preparation process for SBSMA involves the following: (1) placing matrix asphalt in a constant temperature oven set at 140 °C for 3 hours to allow it to flow; (2) heating the matrix asphalt to approximately 180 °C in a heating sleeve, followed by the addition of SBS while stirring, and shearing at a high speed of 4000–5000 rpm until uniform; (3) adding solubilizer and stirring continuously for 4 hours to achieve a stable system.

## 3. Results and Discussion

### 3.1. Analysis of the Conventional Performance of Asphalt

#### 3.1.1. Analysis of Penetration

**Figure 3:**
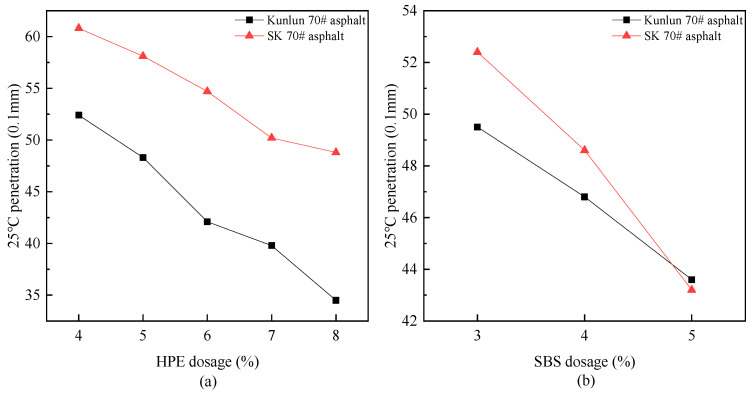
The variation of 25 °C penetration with different modifier ((**a**) PE composite modifier, (**b**) SBS) dosages.

According to the preparation plan for high-performance waste plastic composite modified asphalt, as outlined in the “Test Regulations for Asphalt and Asphalt Mixtures in Highway Engineering” (JTG E20-2011) [44], various concentrations of high-performance waste plastic composite modifier and SBS were selected. Meanwhile, to ensure the rigor and scientific validity of the research, two types of matrix asphalt were also selected for comparative analysis in the laboratory. The results are summarized in Figure 3.

The degree of penetration, serving as an indicator of conditional viscosity, is referred to as isothermal viscosity. A lower penetration value signifies that the asphalt exhibits a relatively high viscosity. When considering the change in penetration, the incorporation of PE composite modifier and SBS significantly decreased the penetration of asphalt, thereby indicating an increase in its viscosity. The increased viscosity demonstrates that the modified asphalt possesses robust shear resistance under high temperature conditions, thereby characterizing the high temperature stability of asphalt materials to a certain degree.

When the PE composite modifier is added, the penetration decreases with the increases with the increase of its dosage. The overall trend is relatively flat, indicating that during the blending process with asphalt, the dispersion and grinding effect is enhanced through mechanical mixing and high-speed shear, resulting in improved compatibility between the PE composite modifiers and asphalt. For Kunlun A-grade 70# heavy asphalt, the penetration reaches the standard of SBS (I-D) modified asphalt when 4–6% PE composite modifier is added, in accordance with the performance indicators specified in the “Technical Specifications for Construction of Highway Asphalt Pavements”. The average penetration is reduced to 75.6% of its pre-modification level, indicating a significant alteration in the asphalt viscosity. When 5–8% PE composite modifier is added to SK 70# heavy asphalt, the penetration of the modified asphalt meets the requirements of SBS (I-D) modified asphalt. The penetration value of SK Grade A 70# heavy asphalt prior to modification exceeds that of Kunlun Grade A 70# heavy asphalt.

When incorporating 3% to 5% SBS, the modification effect on two types of matrix asphalt is notable. For Kunlun Grade A 70# heavy asphalt, the modification effect achieved with the incorporation of 3% to 5% SBS modifier is comparable to that obtained with the addition of 4% to 6% PE composite modifier. For SK70# asphalt, the modification effect resulting from the inclusion of 3% to5% SBS modifier is more akin to that observed with the incorporation of 6% to 8% PE composite modifier.

As illustrated in Figure 3, the modification effect exhibited by the PE composite modifier on Kunlun 70# asphalt is notably more pronounced, and similarly, the effect of SBS on SK70# asphalt is more significant.

#### 3.1.2. Analysis of Softening Point

**Figure 4:**
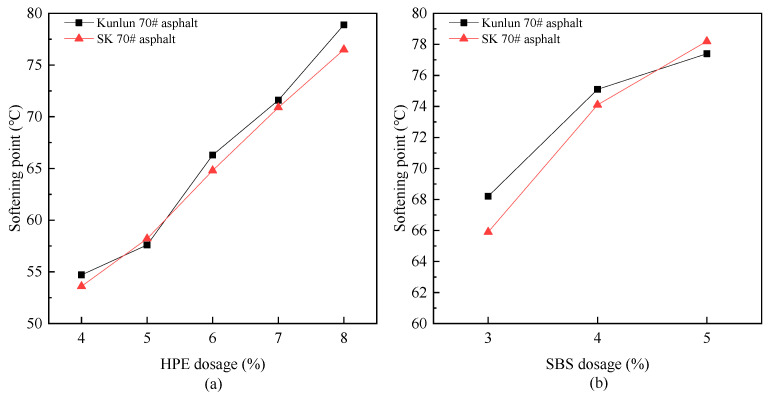
The variation of softening point with different modifier ((**a**) PE composite modifier, (**b**) SBS) dosages.

The softening point is an important indicator that reflects the high-temperature performance of asphalt. In general, the higher the softening point, the better the high-temperature performance of asphalt. A comparative study was conducted to investigate the effects of PE composite modifier and SBS on the softening point of Kunlun 70# asphalt and SK70# asphalt, respectively. The result is shown in Figure 4.

As illustrated in Figure 4a, with the increase of HPE, the softening point of modified asphalt increases. However, the impact on the softening point of different matrix asphalt is not significantly different. When the dosage of HPE exceeds 6%, the softening point meets the requirements for SBS (I-D) modified asphalt in the “Technical Specifications for Construction of Highway Asphalt Pavements”. This is because the particles of the HPE are finely ground by specific process equipment (<10 μm). Upon addition to asphalt, the surface free energy of PE particles decreases, causing them to adsorb structurally similar components in asphalt and undergo adsorption and swelling due to the action of lightweight components. The adsorption and swelling process alters the aggregate composition of asphalt, resulting in increased viscosity, improved the softening point, and enhanced strength and deformation resistance.

Figure 4 demonstrates that the modification effect of 3% SBS on the softening point of asphalt is approximately equivalent to that of 6% HPE, with the modification effect of SBS being more significant. Figure 4b further illustrates that SBS has a more pronounced effect on the softening point of SK 70# asphalt.

#### 3.1.3. Analysis of Ductility

**Figure 5:**
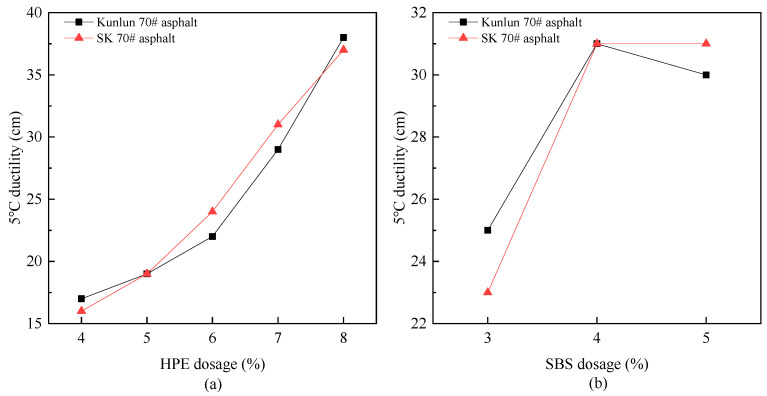
The variation of ductility with different modifier ((**a**) PE composite modifier, (**b**) SBS) dosages.

Ductility serves as an indicator of the low-temperature performance of asphalt. The ductility of modified asphalt with different modifiers was evaluated and the results are presented in Figure 5.

Figure 5 clearly demonstrates that there is a noticeable variation in the effect of the two modifiers on the ductility of asphalt.

From 5a illustrates that the effect of HPE dosage on the ductility of SK70# matrix asphalt and Kunlun 70# matrix asphalt exhibits a comparable impact. When the dosage exceeds a level of 6%, the ductility fulfills the criteria specified for SBS (I-D) modified asphalt in the “Technical Specifications for Construction of Highway Asphalt Pavements”. HPE absorbs wax components from the matrix asphalt and melts under heat application, transforming from a crystalline to an amorphous state. During mechanical mixing, polyethylene is uniformly dispersed into the asphalt, with wax molecules penetrating within the interior of the polyethylene structure, causing swelling and stretching of the polyethylene chains. After cooling, the polyethylene chain recrystallizes, resulting in an increase in its ability to resist external forces. Therefore, this process leads to an enhancement in the viscosity of asphalt and improved low-temperature ductility.

However, when the SBS content exceeds 4%, the elongation of the modified asphalt begins to increase slowly or even decreases slightly. SK70# asphalt demonstrates a significant sensitivity to SBS content, suggesting a specific range of SBS content that is optimal. Therefore, an appropriate SBS content should be selected based on the specific engineering requirements.

### 3.2. Analysis of Storage Stability of Asphalt

**Figure 6:**
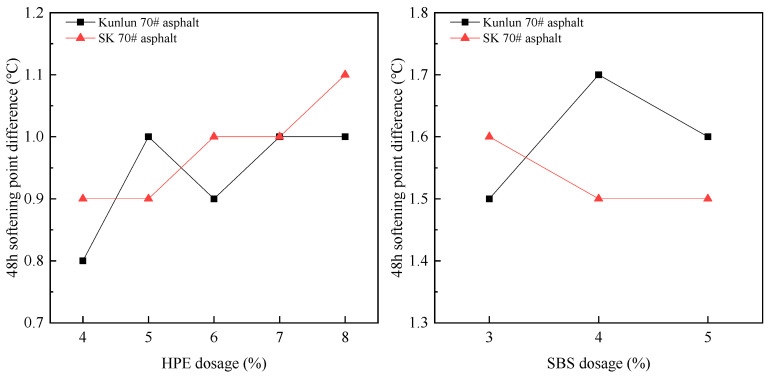
The variation of 48 h softening point difference with different modifier ((**a**) PE composite modifier, (**b**) SBS) dosages.

The assessment of storage stability is crucial for demonstrating the stability of asphalt. In accordance with the standard, the difference in softening points after 48 h of storage is selected as the evaluation criterion. The 48 h softening point difference of HPEA and SBSMA were tested and the results are presented in Figure 6.

From Figure 6, it is evident that there is no significant difference in the 48-h softening point difference of HPEA at various HPE dosages and the overall values are small, indicating that the segregation phenomenon of HPEA is insignificant. Despite the addition of stabilizer and compatibilizer during the modification process, after 48 h, the softening point difference of SBSMA was higher than that of HEPA, indicating the presence of some segregation phenomenon in SBSMA. Consequently, based on the analysis of the softening point difference, HPEA exhibits better storage stability than SBS modified asphalt.

HEPA exhibits good storage stability and no obvious segregation phenomenon. This is attributable to HPE’s exceptional flexibility, elongation, and impact resistance. Furthermore, HPE possesses a relatively high molecular weight and contains numerous alkyl side chains and methyl branches along its long chain, resulting in a multi-branched dendritic structure. Concurrently, the presence of multiple branching and irregular molecular structures significantly enhances the viscosity of asphalt, leading to improved adhesion between modified asphalt and aggregates.

### 3.3. Analysis of Anti-Aging Performance of Asphalt

**Figure 7:**
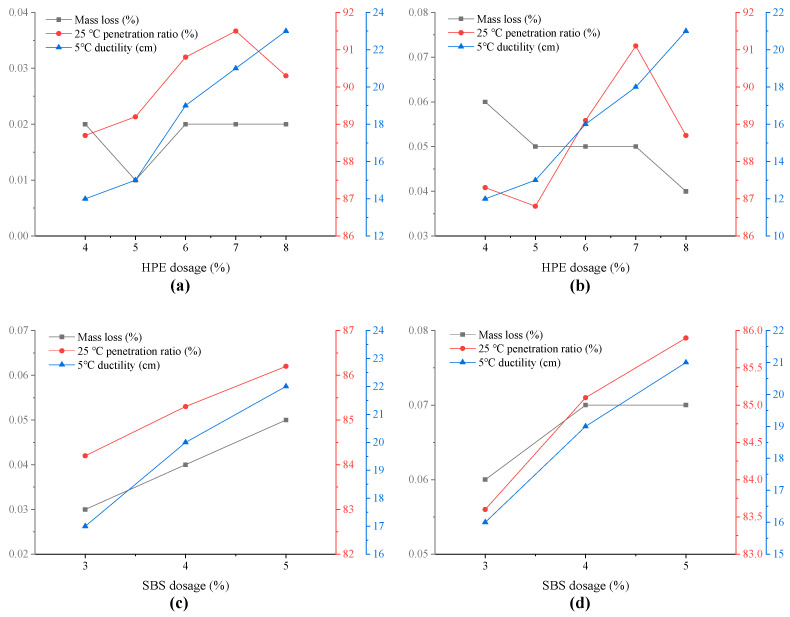
Influence of modifier type and content in different base asphalt on the anti-aging performance index of modified asphalt ((**a**) Kunlun 70# asphalt and HPE, (**b**) SK 70# asphalt and HPE, (**c**) Kunlun 70# asphalt and SBS, (**d**) SK 70# asphalt and SBS).

The thin film oven test (TFOT) was utilized to simulate the thermal oxidative aging of various asphalt samples. A comparative analysis of the basic performance indicators following aging was performed, as illustrated in Figure 7.

As depicted in Figure 7a,b, for two different types of matrix asphalt, with an increase in the dosage of HPE, the mass loss of modified asphalt after aging remains within 0.06%, indicating minimal change. The 5 °C ductility of two types of modified asphalt after aging exhibits an upward trend with an increase in HPE content. This suggests that within a specific range, an increase in HPE content results in improved aging resistance of modified asphalt.

As shown in Figure 7c,d, with the increase of SBS dosage, the 25 °C penetration ratio and 5 °C ductility of the two modified asphalts after aging exhibit an increasing trend. It indicates that within a certain range, a higher dosage of SBS results in better aging resistance of modified asphalt.

When the same matrix asphalt is used, the influence of the two modifiers on the aging resistance of asphalt can be compared. For example, by comparing Figure 7a,b, it can be observed that when HPE reaches a certain content, HPEA can achieve anti-aging performance comparable to or even better than that of SBSMA.

### 3.4. Analysis of the Microscopic Properties of Asphalt

#### 3.4.1. Analysis of Fluorescence Microscopic Test

**Figure 8:**
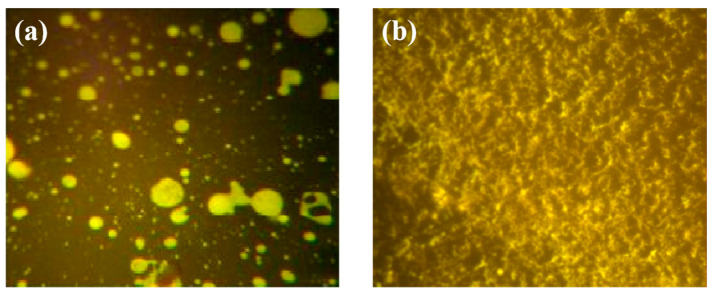
Fluorescence microscopy image of HPEA ((**a**) before adding stabilizer, (**b**) after adding stabilizer).

Optical microscopy is an effective auxiliary analytical tool for studying the thermal stability of polymer modified asphalt. Recently, micrographs have been used as a direct method to study the distribution behavior and phase interface behavior of polymers in asphalt systems. The dispersibility of modifiers in asphalt was evaluated by directly observing the distribution of polymers in asphalt [41]. HPEA was prepared using Kunlun 70# asphalt at a 6% HPE content. The microstructure of HPEA before and after the addition of stabilizer was observed using a fluorescence microscope, as shown in Figure 8.

Figure 8 depicts the fluorescence microscopy image of HPEA, with the yellow material signifying the plastic particle matrix and the black-shaded area denoting the asphalt matrix. Figure 8a illustrates that following a period of high-temperature and high-speed shearing, HPE is predominantly uniformly dispersed. However, some larger HPE particles remain randomly dispersed at various locations within the asphalt. This suggests that during the preparation process of HPEA, HPE particles undergo swelling in asphalt and become disperse through shear forces. As depicted in Figure 8b, following the addition of stabilizer for development, the HPEA system achieves a high degree of uniformity, with no larger particles present. This suggests that the stabilizer exerts a favorable effect on the dissolution and dispersion of HPE within asphalt. Upon the addition of the stabilizer, HPE undergoes development and re-crosslinks within the system, leading to smaller particles interacting with asphalt to form a relatively stable three-dimensional spatial structure. This accounts for the high-temperature stability of HPEA, which manifests macroscopically as a high softening Concurrently, larger particles coalesce following high-temperature melting, enhancing the low-temperature flexibility of HPEA, which manifests macroscopically as increased ductility.

#### 3.4.2. Analysis of Infrared Spectrum Test

**Figure 9:**
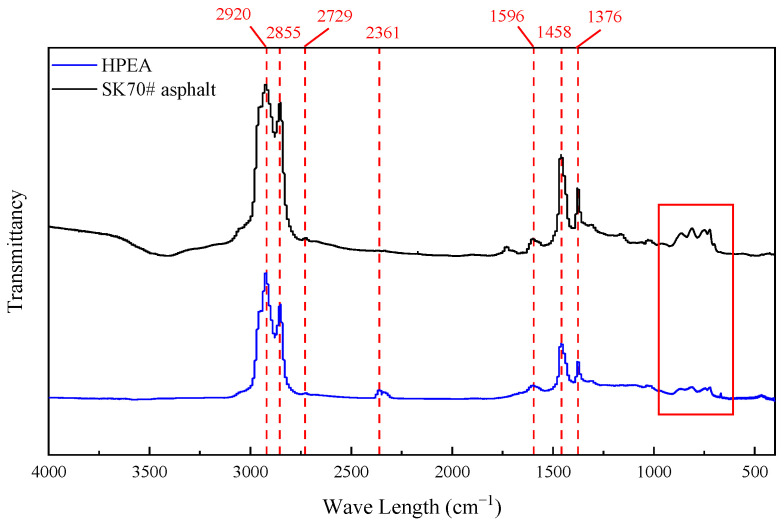
The infrared spectra of different types of asphalt.

Based on the results obtained from the analysis of fluorescence microscopic test, infrared spectroscopy analysis was conducted on the matrix asphalt and HPEA and the results are presented in Figure 9.

As illustrated in Figure 9, the asphalt samples exhibit prominent absorption peaks at 2800 cm^−1^~3000 cm^−1^, which are attributed to the CH_2_ stretching vibration absorption peak of alkanes or cycloalkanes. A weak absorption peak at 2729 cm^−1^ corresponds to the C-H stretching vibration. The infrared spectral peaks of polymers typically occur in two distinct regions: 4000 cm^−1^~1300 cm^−1^ and 1300 cm^−1^~600 cm^−1^. The vibration absorption effect of functional groups is particularly pronounced in the high-frequency region, which aids in analysis and is crucial for identifying functional groups. The low-frequency region is highly sensitive to asphalt components and minor changes can result in a significant vibration absorption effect. Therefore, this region is often referred to as the fingerprint recognition area [42,43]. At the wavelength of 2361 cm^−1^, HPEA exhibit vibrations, indicating the presence of asymmetric vibrations associated with accumulated double bonds or stretching vibrations of triple bonds such as –C≡C and –C≡N. However, there is no absorption peak in the SK70# matrix asphalt at this wavelength, indicating the formation of new chemical bonds during the preparation of HPEA. The absorption peaks of HPEA and SK70# asphalt at 1458 cm^−1^ and 1376 cm^−1^ are formed by the in-plane stretching vibration of –C-H in –C-CH_3_ and –CH_2_.

There exists a significant difference in the absorption peaks between HPEA and SK70# asphalt in the fingerprint region. In the 1000–650 cm^−1^ region, a benzene ring substitution zone exists, which results in a benzene ring skeleton (C-C) vibration and bending vibration (C-H). The difference in absorption peaks in the fingerprint region is primarily attributed to the slight modifications in molecular structure induced by the incorporation of HPE. The shapes of the spectra of HPEA and SK70# asphalt are largely similar, suggesting that the modification of HPEA predominantly entails physical blending.

## 4. Conclusions

HPEA and SBSMA were prepared and a comparative analysis was conducted on the conventional performance, storage stability, and aging resistance. The microscopic characteristics of HPEA were examined by using FM and FTIR. The main conclusions are summarized as follows:

(1) The conventional performance of HPEA is comparable to, or superior to, that of SBSMA. The addition of HPE resulted in a significant decrease in asphalt penetration. The modification effect achieved by adding 3–5% SBS to Kunlun 70# asphalt is equivalent to that achieved by incorporating 4–6% HPE. As the concentration of high-performance waste plastic composite modifiers increases, the ductility of the modified asphalt gradually enhances. When the dosage of HPE exceeds 6%, the ductility of HPEA fulfills the criteria for SBS (I-D) modified asphalt specified in the standard.

(2) HEPA exhibits good storage stability and no obvious segregation phenomenon. This is attributed to HPE’s exceptional flexibility, elongation, and impact resistance. Furthermore, HPE possesses a relatively high molecular weight and contains numerous alkyl side chains and methyl branches along its long chain, contributing to its multi-branched dendritic structure.

(3) When HPE attains a specific concentration, HPEA can exhibit an anti-aging performance that is comparable to, or superior to, that of SBSMA.

(4) Upon examination using FM and infrared spectroscopy, it was observed that HPE swells and uniformly disperses within asphalt after undergoing high-temperature and high-speed shearing. The infrared spectrum of HPEA closely resembles that of SK70# matrix asphalt. This suggests that the modification of HPEA primarily involves physical blending, with HPE undergoing development and re-crosslinking within the system, leading to interactions between smaller particles and asphalt, resulting in the formation of a relatively stable three-dimensional spatial structure.

## Figures and Tables

**Table 1:** Technical index and test data of 70# matrix asphalt.

Project		Index	Test Result
135 °C Brinell viscosity (Pa·s)		/	0.56
Penetration (0.1 mm) (25 °C, 100 g, 5 s)		60~80	68.7
Softening point (℃)		≥46	50.0
Ductility (5 cm/min,15 °C)		≥100	>100
Ductility (5 cm/min,10 °C)		≥20	80.7
After TFOT	Mass loss (%)	≤±0.8	0.49
	25 °C, penetration ratio (%)	≥61	65.7
Flash point (°C)		≥260	315
Bitumen relative density (g/cm^3^)		Field record	1.029

**Table 2:** Basic properties of SBS.

Properties	Unit	Value
S/B ratio	/	30/70
Extender oil content	/	0
Modulus at 300%	MPa	≥2.2
Tensile strength	MPa	≥16.0
Elongation at break	%	≥700
Tensile set at break	%	≤40
Melt flow rate	g/10 min	0.5~2.5
Molecular weight	/	100,000

## Data Availability

Data are contained within the article.
